# Crowdsourcing and the feasibility of manual gene annotation: A pilot study in the nematode *Pristionchus pacificus*

**DOI:** 10.1038/s41598-019-55359-5

**Published:** 2019-12-11

**Authors:** Christian Rödelsperger, Marina Athanasouli, Maša Lenuzzi, Tobias Theska, Shuai Sun, Mohannad Dardiry, Sara Wighard, Wen Hu, Devansh Raj Sharma, Ziduan Han

**Affiliations:** Max Planck Institute for Developmental Biology, Department for Integrative Evolutionary Biology, Max-Planck-Ring 9, 72076 Tübingen, Germany

**Keywords:** Evolutionary genetics, Phylogenetics, Computational biology and bioinformatics, Genome informatics

## Abstract

Nematodes such as *Caenorhabditis elegans* are powerful systems to study basically all aspects of biology. Their species richness together with tremendous genetic knowledge from *C. elegans* facilitate the evolutionary study of biological functions using reverse genetics. However, the ability to identify orthologs of candidate genes in other species can be hampered by erroneous gene annotations. To improve gene annotation in the nematode model organism *Pristionchus pacificus*, we performed a genome-wide screen for *C. elegans* genes with potentially incorrectly annotated *P. pacificus* orthologs. We initiated a community-based project to manually inspect more than two thousand candidate loci and to propose new gene models based on recently generated Iso-seq and RNA-seq data. In most cases, misannotation of *C. elegans* orthologs was due to artificially fused gene predictions and completely missing gene models. The community-based curation raised the gene count from 25,517 to 28,036 and increased the single copy ortholog completeness level from 86% to 97%. This pilot study demonstrates how even small-scale crowdsourcing can drastically improve gene annotations. In future, similar approaches can be used for other species, gene sets, and even larger communities thus making manual annotation of large parts of the genome feasible.

## Introduction

How well can biological knowledge be transferred across species? Are biological functions carried out by the same genes in different organisms? How fast do regulatory networks diverge? In order to address these fundamental questions, more than 20 years ago, the nematode *Pristionchus pacificus* has been introduced as a so-called “satellite” model organism to one of the most successful animal model systems, *Caenorhabditis elegans*^1,2^. Since then, several comparative studies in developmental and ecological contexts have highlighted the importance of developmental system drift as a concept in evolution^3^ and have demonstrated that the divergence between *Pristionchus* and *Caenorhabditis* was accompanied by extensive chemical^4–6^, genic^7–9^, and morphological^10–12^ innovations. The establishment of multiple genetic^13,14^ and genomic tools and resources^15,16^ by Sommer and colleagues motivated an increasing number of independent groups to adapt *P. pacificus* as a model system for comparative studies at a mechanistic level^17–21^. However, reverse genetic approaches based on candidate genes with known functions in *C. elegans*^22,23^ have been hampered not only by the huge amount of lineage-specific duplications^23–26^, but also by missing and incorrect gene annotations. Traditionally, protein-coding genes are annotated by gene prediction algorithms that model general sequence features of transcription and translation start and end sites, as well as splicing signals^27–29^. This can be complemented with evidence based approaches using transcriptomic and protein homology data^30,31^. While automated annotation pipelines perform reasonably well to be useful for genetic screens^32–34^ and evolutionary genomic analyses^35–37^, their outcomes by far do not meet the standards of the gene annotations from classical model organisms such as *C. elegans*, *Drosophila melanogaster*, and *Mus musculus* that have been curated over decades by a large research community^38^. In order to make the *P. pacificus* system more tractable for researchers without extensive genomic and phylogenetic expertise, we need to minimize the discrepancy in gene annotation quality between *C. elegans* and *P. pacificus*. To this end, we employed an integrative approach using comparative genomic and transcriptomic data combined with crowdsourcing to improve the *P. pacificus* annotations of *C. elegans* homologs and orthologs. First, we carry out a comparative assessment of 22 nematode genomes and demonstrate that *P. pacificus* has one of the best available nematode genomes. Second, we perform a genomewide screen for *C. elegans* genes where homologs and orthologs are not or incorrectly annotated in the *P. pacificus* genome. Third, a community-based manual curation of suspicious gene models reveals thousands of hidden orthologs and missing homologs. This pilot study can be extended to even larger gene sets and communities possibly employing citizen scientists, which would raise the quality of gene annotations to the next level^38^.

## Results

### The quality of nematode draft genomes is highly heterogeneous

**Figure 1:**
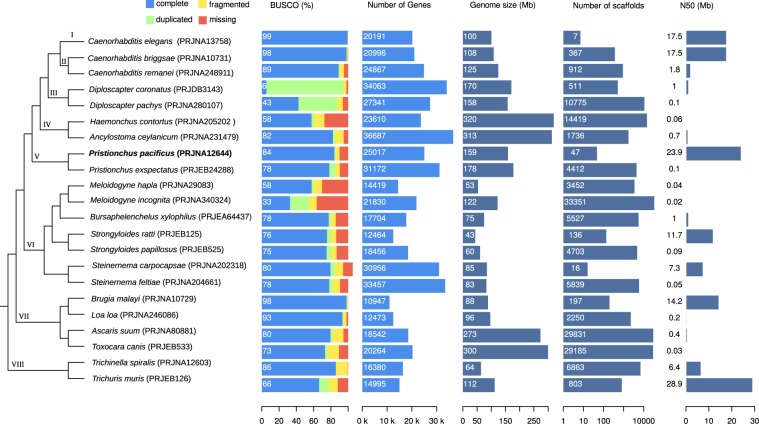
Comparative assessment of nematode genome quality. Genomic data for 22 nematode species was obtained from WormBase ParaSite (release WBPS13) and evaluated based on completeness level of gene annotations and genome assembly contiguity. The barplots show the results of a benchmarking of single copy orthologs (BUSCO^40^) analysis, the number of genes, genome sizes, number of scaffolds, and the N50 measure of assembly contiguity. The genome and annotations of *P. pacificus* exhibit an overall comparatively high quality. The schematic phylogeny is based on phylogenomic analysis of 108 nematodes^39^, Roman numerals indicate phylostrata that are used for further analysis.

To obtain a general overview of the current status of nematode genome quality, we analyzed assemblies and gene annotations of 22 species (Fig. 1). The species were arbitrarily selected to span the diversity of the nematode phylum^39^. We will further use this taxon sampling to perform an analysis of gene age, i.e. phylostratigraphic analysis where each phylostratum is defined by at least two outgroup species to minimize the effect of species-specific gene loss. Nematode genomes range in size between 43 and 320 Mb and contain between 11 and 37 thousand annotated protein-coding genes (Fig. 1). Analyses of assembly features and gene annotations indicate a wide range of qualitative variability. Some genomes are assembled and scaffolded to the level of chromosomes with high degrees of contiguity (the N50 value which is a measure of genome assembly contiguity is up to 29 Mb) whereas others are largely fragmented into up to 33 thousand scaffolds with N50 values below 0.1 Mb (Fig. 1). Similarly, analyses of completeness levels based on benchmarking univeral single copy orthologs (BUSCO^40^) reveal substantial amount of either missing or duplicated genes and it is not totally clear to what extent these differences are of biological or technical nature^41^. In the case of *Diploscapter coronatus*, the apparent high fraction of duplicated genes could either be explained by hybridization of two divergent lineages or a whole genome duplication^42^. The genome of *P. pacificus*, which was generated by assembly from single-molecule, long-read sequencing data and scaffolding with the help of a genetic linkage map^15^, shows one of the highest levels of contiguity (47 scaffolds, N50 = 24 Mb). Gene annotations were generated by the MAKER2 pipeline^30,31^ which combined gene prediction algorithms, transcriptome data, and protein homology data from other *Pristionchus* species^11,15,43^. The completeness level of gene annotations (BUSCO completeness: 84%) is in the upper range when compared to most other nematode genomes (median 78%, interquartile range (IQR): 68–85%, Fig. 1). This demonstrates the relatively high quality of the current *P. pacificus* assembly and gene annotations.

### Complementary genome and transcriptomes reveal potentially missing gene models

The completeness analysis as implemented in the software BUSCO^40^ can also be applied to the raw genome assembly of *P. pacificus*. This yielded a combined completeness value of 93% (complete single copy and duplicates) as compared to 86% for the *P. pacificus* gene annotations and indicates towards the presence of incorrectly annotated or missing *C. elegans* orthologs in the genome of *P. pacificus*. Moreover, the fact that a recent *de novo* transcriptome assembly that was based on a strand-specific RNA-seq data set exhibited an even higher combined completeness level of 97% (Table 1) demonstrates even further room for improvement^16^. Finally, single-molecule, long-read transcriptome sequencing data were recently generated for *P. pacificus* which allows a much more accurate definition of gene structures from reference alignments of single reads^44^. However, neither transcriptomic data set was available when the existing gene annotations (version: El Paco annotation v1/WormBase release: WS268) were generated and they could still be used for further improvement.

**Table 1:** Completeness analysis of different *P. pacificus* data set.

Data set	BUSCO (%)				Ref
	Complete Single Copy (+Duplicates)	Duplicate	Fragmented	Missing	
Genome assembly (El Paco assembly)	91.6 (92.9)	1.3	3.1	4.0	^15^
El Paco annotation v1/WS268	84.0 (85.8)	1.8	4.3	9.9	^15^
*de novo* transcriptome assembly	59.1 (97.1)	38.0	2.6	0.3	^16^
Iso-Seq assembly	48.0 (73.3)	25.3	10.9	15.8	^44^
El Paco annotation v2	95.4 (97.1)	1.7	2.0	0.9	this study

The high level of duplicates in the two transcriptomic data sets is due to the presence of isoforms.

**Figure 2:**
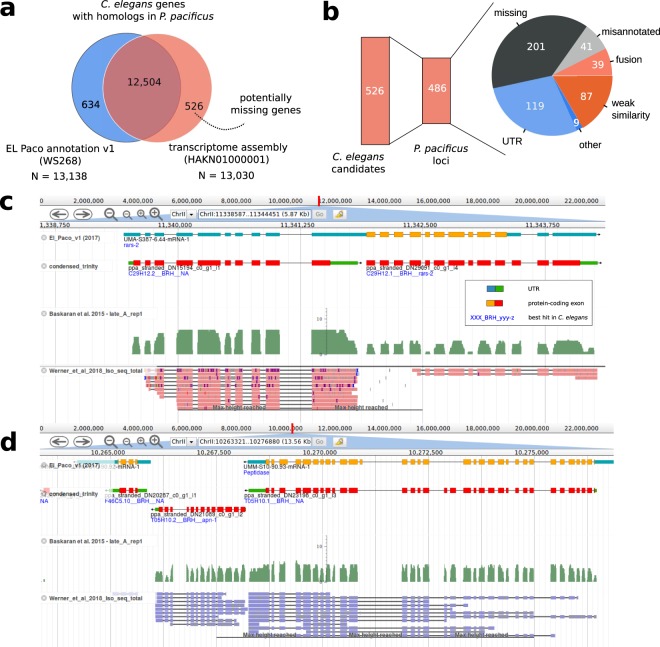
Identification of missing genes. (**a**) 526 potentially missing genes were identified based on *C. elegans* genes with homologs in the transcriptome assembly but not in current gene annotations. (**b**) The 526 missing gene candidates were located in 486 *P. pacificus* loci that were classified based on community annotators. (**c**) The genome browser screenshot shows a homolog of *C. elegans* C29H12.2 which is located in the annotated 5′UTR of a *P. pacificus* gene. This locus harbors two *P. pacificus* transcripts with different expression levels and well supported as non-overlapping transcripts based on RNA-seq and Iso-seq data. (**d**) A homolog of apn-1 is completely missing from current gene annotations.

To systematically identify potentially missing genes in the *P. pacificus* genome, we searched for *C. elegans* genes lacking homologs in the current *P. pacificus* gene annotations (BLASTP e-value < 10^−5^) but having a matching open reading frame in the *de novo* transcriptome assembly (Fig. 2a). While 12,504 (62%) *C. elegans* genes had BLASTP hits in both data sets, 634 (3%) *C. elegans* genes showed only BLASTP hits against the current gene annotations suggesting that these genes are properly annotated but are expressed so weakly that they were not captured in the transcriptome assembly of mixed-stage cultures^45^.

Similarly, we identified 526 (3%) *C. elegans* genes that were only found in the transcriptome assembly and therefore represent candidates for missing gene annotations.

### Community-based curation identifies missing genes in the ***P. pacificus*** genome

In order to improve the existing gene annotations, we chose to manually inspect and classify all 526 missing gene candidates in the *P. pacificus* genome browser (http://www.pristionchus.org). Thereby, we recruited and trained colleagues as community annotators, who would be capable to classify a genomic locus and to propose a correction to the existing gene models (see *Methods*). Lists of missing gene candidates were shared in online spreadsheets and documents, which allowed multiple annotators to inspect and correct candidate loci in parallel. 119 (25%) of the 486 non-redundant *P. pacificus* loci were classified as missing genes in predicted UTRs of annotated genes (Fig. 2b). We would speculate that this is caused by the fact that nematode genomes are compact and UTR regions can frequently overlap^45^. This can cause artificial fusion of transcripts during the assembly of RNA-seq data. Consequently, only the largest ORF of such a gene is annotated as protein-coding and the rest is classified as 3′ and 5′ UTR. Alternatively, this problem could arise when a fused gene prediction from the sister species is used as homology information but MAKER2 fails to generate a complete gene model out of it. The *C. elegans* gene C29H12.2 is one example of a missing gene model residing in the UTR of a *P. pacificus rars-2* homolog (Fig. 2c). The corresponding *P. pacificus* locus is spanned by two assembled transcripts that are homologous two C29H12.2 and *rars-2*, respectively. Both transcripts are also well supported by Iso-seq data and exhibit different expression levels^44,46^. In such a case, we would propose a replacement of the old *P. pacificus* gene model by the two distinct transcripts.

After manual inspection of all 526 missing gene candidates, 201 (41%) of the 486 non-redundant *P. pacificus* loci were classified as missing genes (Fig. 2b). Presumably this kind of error could arise when the gene annotation pipeline is mostly dependent on gene prediction algorithms which fail to predict all genes in gene dense regions (e.g. operon structures) as the intergenic distances might span only a few hundred nucleotides, which could be too small for triggering the initiation of a new gene model. The *C. elegans* gene *apn-1* is one example of a missed gene model in a gene dense region (Fig. 2d). Given that the *P. pacificus* homolog of *apn-1* has good transcriptomic support, the correction in this case would simply add the transcript to the existing gene models. Other instances of missing homologs are due to borderline cases in the BLASTP searches where one search resulted in an e-value slightly below the e-value threshold (10^−5^) and the result of the other BLASTP search was slightly above the threshold. In total, we encountered 87 of such cases which we termed ‘weak similarity’ (Fig. 2b). For such cases no correction was proposed. In summary, we compiled corrections for 280 *P. pacificus* genes which were replaced by 714 new gene models. All these changes were submitted to WormBase and were incorporated in the release WS272.

### Artificial gene fusions mask thousands of hidden orthologs

**Figure 3:**
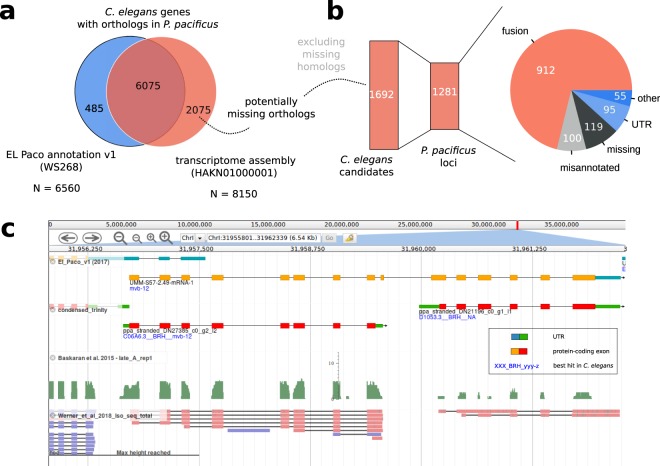
Community-based curation of hidden orthologs. (**a**) We identified 2075 putative *C. elegans* one-to-one orthologs that were specific to the *P. pacificus* transcriptome assembly. (**b**) Community-based curation classified most of the corresponding gene loci as artificial gene fusions. (**c**) Non-overlapping transcripts corresponding to *P. pacificus* orthologs of *mvb-12* and D1053.3 are artificially fused in a current gene model. This prohibits the detection of a one-to-one ortholog of D1053.3 based on a genome-wide approach such as best reciprocal hits.

A small number of *C. elegans* genes with missing homologs in the current gene annotations (version: El Paco v1/WS268) of *P. pacificus* were classified as located in fused gene models (Fig. 2b). One potential explanation could be that an artificially fused gene prediction from the sister species is taken as homology data to annotate the orthologous locus in *P. pacificus*, but small errors cause parts of the gene model to be either incompletely or incorrectly annotated in *P. pacificus* resulting in a loss of detectable homology (Fig. 2c). Even if the homolog of a *C. elegans* gene is incorporated in the correct ORF within an artificially fused gene model, this could still cause a loss of one-to-one orthology as the corresponding *P. pacificus* gene can only be identified as one-to-one ortholog of a single *C. elegans* gene. Thus, we performed a second screen for *C. elegans* genes that had a predicted one-to-one ortholog (best-reciprocal hit) in the transcriptome assembly but not in current gene annotations (Fig. 3a). In total, 6075 (93%) of *C. elegans* genes with a predicted one-to-one ortholog (based on best-reciprocal hits) in current gene annotations, also had a predicted one-to-one ortholog against the *de novo* transcriptome assembly (Fig. 3a). Nevertheless, we found 2075 *C. elegans* genes that only had predicted one-to-one orthologs in the *de novo* transcriptome assembly. Excluding *C. elegans* genes that were identified already in the previous screen for missing homologs, this resulted in 1692 *C. elegans* genes with predicted one-to-one orthologs in the *de novo* transcriptome assembly but not in the current set of gene annotations (version: El Paco v1/WS268). Community-based classification and curation of the 1281 corresponding *P. pacificus* loci classified 912 (71%) cases as artificial gene fusions (Fig. 3b). One such an example is the *C. elegans* gene D1053.3. Its putative ortholog is fused with the *P. pacificus mvb-12* ortholog (Fig. 3c). Apart from being orthologous to two different *C. elegans* genes, both *P. pacificus* genes are supported as non-overlapping transcripts by RNA-seq and Iso-seq, and are expressed at different levels. This confirmed the interpretation of an artificially fused annotation. The proposed correction in this case would be a replacement of the old gene model by the two non-overlapping transcripts. In total, we updated 1241 *P. pacificus* gene models and replaced them with 3305 new models. These updates were submitted to WormBase and will be released following curation. The new *P. pacifcus* gene annotation (version: El Paco v2) with 28,036 gene models is also available on http://www.pristionchus.org/download. The results of the BUSCO analysis (Complete and Single Copy: 95.4%, Duplicated: 1.7%, Fragmented: 2.0%, Missing: 0.9%) indicate that the new annotation represents a substantial improvement over the previous annotations^15^ (Table 1).

### Improved gene annotations facilitate the establishment of a catalog of *C. elegans* homologs and orthologs in the *P. pacificus* genome

Since our primary focus was to improve the annotation of *C. elegans* orthologs in the *P. pacificus* genome, we wanted to use the updated gene annotation to generate a catalog of predicted orthologs between *C. elegans* and *P. pacificus*. As the identification of orthologs typically requires sufficient genomic and phylogenetic knowledge to retrieve relevant protein data sets and to perform reconstruction of gene trees^24,45,46^, a genome-wide catalog of orthologs would be highly useful as a starting point for researchers without sufficient expertise. Previous comparisons between *C. elegans* and *P. pacificus* identified putative one-to-one orthologs for roughly 6000–8000 genes^44,46^. To further characterize *C. elegans* genes without orthologs in *P. pacificus*, we additionally carried out a phylostratigraphic analysis^47^ to estimate their relative age. Basically, phylostratigraphy uses absence-presence patterns of a gene to map its origin to an internal branch in a species tree^47^. Our analysis revealed that 5258 (26%) of *C. elegans* genes do not have BLAST hits in *Pristionchus* or more distantly related species (Phylostrata I–IV, Supplemental Table 1). This strongly suggests that they are younger than the common ancestor between *C. elegans* and *P. pacificus* and consequently have no orthologs. Next, we applied two different approaches to predict orthologs between *C. elegans* and *P. pacificus*: best reciprocal hits and Markov clustering as implemented in the software orthAgogue^48^. Computation of best reciprocal hits is a standard approach for predicting one-to-one orthologs across species^49,50^. In order to capture more complex orthology relationships (e.g. many-to-many), more general approaches such as Markov clustering have been widely applied^48,51^. Based on best reciprocal hits, we identified 8348 predicted one-to-one orthologs between both species (Supplemental Table 1) whereas the orthAgogue pipeline identified 7643 orthologous clusters, of which only 3345 corresponded to one-to-one orthologs. The large majority (98%) of these predicted one-to-one orthologs was also identified as best reciprocal hits and in 3260 (99%) cases, the same *P. pacificus* gene was predicted as one-to-one ortholog. The large discrepancy between the total number of best reciprocal hits and one-to-one orthologs defined by orthAgogue could be explained by the fact that best reciprocal hits do not take inparalogs into account^49^. However, only 1049 (21%) of *C. elegans* genes that were not identified as one-to-one orthologs by orthAgogue could be explained by the presence of lineage-specific inparalogs, suggesting that orthAgogue with default settings might be too conservative for this analysis. This is further supported by the reanalysis of 57 one-to-one orthologous pairs that were previously confirmed by phylogenetic analysis^46^. While 53 of the previously confirmed one-to-one orthologs were captured as best reciprocal hits, only 33 were also identified by orthAgogue. Taken together, the improved gene annotation facilitated the prediction of substantially more one-to-one orthologs (Fig. 3a, Supplemental Table 1). This resource can be taken as a starting point to identify candidate genes in *P. pacificus*.

## Discussion

With *C. elegans*, *C. briggsae*, and *P. pacificus*, three genetically tractable and free living nematode model organisms have been well established and can be used to study the evolution of gene function at various time-scales^2,3,52^. For example, recent reverse genetic approaches in *P. pacificus* have revealed functional divergence of genes with known roles in *C. elegans* dauer formation^22,23,53^. In addition, mutant screens in *P. pacificus* for social behaviours have uncovered multiple orthologous *C. elegans* genes for which a behavioral phenotype had been overlooked previously^33,54^. Together with complementary studies of the functional importance of novel genes^7,32,55^, this makes nematodes an extremely powerful system to study genome evolution and gene function at a mechanistic level.

In order to facilitate fruitful functional studies across multiple model organisms, it is crucial to generate genomic resources (e.g. assemblies, annotations) and experimental genetic toolkits (e.g. forward and reverse genetics) of comparable quality. The chromosome-scale assembly of the *P. pacificus* genome^15^ was a major step towards making this species more tractable for other groups. In our study, we aimed to minimize the discrepancy between automatically generated gene annotations for *P. pacificus* and heavily curated annotations for *C. elegans*. To this end, we incorporated recently generated Iso-seq and RNA-seq data into current gene annotations by manual curation of suspicious candidate loci that were identified by comparative genomic analysis. While application of alternative annotation pipelines can generate overall better gene annotations^29,41^, they cannot guarantee that gene annotations will only improve. In certain cases, new annotation pipelines will also cause new errors. In contrast, during manual inspection, each community curator has the choice to not propose any change of gene models in case of uncertainty. Thus, manual inspection should only lead to removal of errors and thus improve annotation quality without introducing biases elsewhere. While manual annotation is an incredibly tedious task that is probably not scalable to complete genomes^38^, we minimized the workload by focusing on a small gene set of *C. elegans* orthologs, recruiting colleagues as community curators, and restricting the task just to the selection of alternative gene models that were generated from transcriptomic data^16,44^ or previous rounds of gene prediction^56,57^. In our opinion, the most crucial aspect of this community project is a good training of new annotators. We achieved this by personal training sessions between experienced and new annotators and the possibility to always discuss cases of uncertainty with other curators. For larger projects, initial training could be achieved by comprehensive online tutorials and communication via email, but this will likely be less efficient. In the case of the *P. pacificus* gene annotations, our study raised the gene count from 25,517 to 28,036 and increased the single copy ortholog completeness level from 86% to 97%. In the *P. pacificus* genome, the greatest source of error was the artificial fusion of neighboring genes. This type of error might be more prevalent in nematodes where genomes are compact^9^ and genes frequently overlap^37,45^. Consequently, manual annotation of restricted gene sets has been proposed and applied previously to circumvent this problem^58^. Given that nematode genomes tend to be pretty compact (Fig. 1), we anticipate that misannotation due to overlapping gene models should be much less pronounced in large vertebrate or plant genomes. Nevertheless, it would be interesting to apply similar screens for gene annotation artifacts to other systems and eventually this could reveal some incorrect annotations in the genomes of classical model organisms.

While this study was restricted to *P. pacificus* genes with putative orthologs in *C. elegans*, we cannot reliably estimate the fraction of erroneous gene models across the whole genome. Our results would suggest that the fraction of missing genes is around one percent (Fig. 2a,b) and the amount of gene models affected by artificial fusions may be up to 15% (Fig. 3a). However, as the *P. pacificus* genome has a higher gene density and a higher concentration of old genes at the chromosome centers^8,15^, we hypothesize that errors due to artificial gene fusions should be much less pronounced at chromosome arms. To test this, an unbiased quantification of error rates across genomic segments would be needed. In future, we also plan to focus on large gene families and lineage-specific orphan genes^55^ that were not explicit subjects of this study. Artificial fusions in these classes of genes could be identified by screens for unexpectedly long gene models or unusual protein domain content. For orphan genes abundant RNA-seq studies of different developmental stages^22,46^, tissues^10,46^, environmental conditions^59^, sexes^16^, and genetic backgrounds^60,61^ could be used to detect non-overlapping transcripts that exhibit anticorrelated expression within a single locus. Thus, while our study has demonstrated that community-based curation of gene annotations is feasible and can lead to substantial improvements, continued effort is needed to lift its quality to a level that would be similar to classical model organisms.

## Methods

### Comparative assessment of nematode genomes

We downloaded 22 nematode genomes and corresponding protein sequences from WormBase ParaSite (release WBPS13). For *Steinernema carpocapsae*, the latest version at WBPS14 was used. In case of multiple isoforms, we selected the longest isoform for further analysis. We ran BUSCO (version 3.0.1) in protein mode (option: -m prot) against the nematode_odb9 data set (N = 982 genes) to evaluate the completeness level of available protein sequences.

### Genome browser integration of transcriptomic resources

To allow community annotators to propose alternative gene models, we integrated recent raw read alignments and reference guided transcript assemblies of Iso-seq data^44^ and a *de novo* assembly of strand-specific RNA-seq data^16^ into our genome browser (implemented in jbrowse^62^) on our webserver (http://www.pristionchus.org). Genomic coordinates for the *de novo* transcriptome assembly were generated by alignment to the *P. pacificus* reference genome (version El Paco) with the program exonerate^63^ (version: 2.2.0, options: -m est2genome – dnawordlen 20 – maxintron 20000). To reduce the complexity of this data set, a condensed version of the *de novo* transcriptome assembly (selection of the isoform with the longest ORF as single representative isoform per gene, minimum peptide length of 60 amino acids, removal of single exon transcripts) with annotated best-reciprocal hits and best hits (BLASTP, e-value < 10^−5^) in *C. elegans* was also incorporated into our jbrowse instance. In addition, predicted protein sequences of previous versions of *P. pacificus* annotations (Hybrid1^56^ and TAU2011^57^) were mapped against the *P. pacificus* assembly by exonerate (version: 2.2.0, options: -m protein2genome – dnawordlen 20 – maxintron 20000). All data sets are available under the gene annotation track of the El Paco reference assembly in our genome browser. To evaluate the quality of the two recent transcriptome assemblies, we ran BUSCO (version 3.0.1, options -m trans) against the nematode_odb9 data set (N = 982 genes) for completeness assessment (Table 1).

### Identification of missing and fused gene models in current gene annotations

We ran bidirectional BLASTP (e-value < 10^−5^) searches between *C. elegans* (version: WS260, longest isoform per gene) and two different *P. pacificus* data sets: the annotated proteins (version: El Paco v1, WS268) and the *de novo* transcriptome assembly^16^. For the *de novo* transcriptome, we reduced the redundancy resulting from different isoforms by selecting the longest ORFs per gene. Based on the different BLASTP searches, we first screened for *C. elegans* proteins with BLASTP hits against ORFs in the *de novo* transcriptome assembly but not against the currently annotated proteins. This yielded 526 candidate genes. In a second phase, we screened for *C. elegans* proteins with putative orthologs, defined by best-reciprocal BLASTP relationships, in the *de novo* transcriptome assembly but not in the annotated proteins, resulting in 2075 candidate genes.

### Community-based manual curation of candidate loci

All *C. elegans* genes together with their candidate homologs and orthologs in the *P. pacificus de novo* transcriptome assembly were stored in a shared online spreadsheet. Community annotators were trained to find the corresponding locus in the genome browser by entering the transcript identifier and to manually inspect the surrounding regions that were defined by the encompassing *P. pacificus* gene model. The candidate locus was then classified as untranslated region (UTR) (the query transcript overlapped exons that were annotated as UTR), missing gene (the query transcript did not overlap any annotated exon), gene fusion (the query transcript did overlap protein-coding exons and homology was detected by BLASTP), misannotation (the query transcript did overlap protein-coding exons but no BLASTP hit was found due incorrect reading frame annotation or minimal overlap) or inconclusive. After classification, a correction was proposed that either added new genes (identifiers could be selected from the *de novo* assembled transcripts, Iso-seq assemblies, or previous versions of gene annotations) or replaced an existing gene model by one or more new genes. In such a case the objective was to lose as little annotated coding sequence as possible. Thus, new genes were selected from the above mentioned data set in order to cover as much coding sequence of the initial gene model as possible. If parts of the old gene model were not covered, BLAST searches against *C. elegans* and other *Pristionchus* species were used to split the old gene model into several parts with sequence matches to distinct *C. elegans* genes, or to extract partial protein sequences of the old gene model that were not covered. Such protein sequence stretches were given a pseudo identifier and were stored in a shared online document. All these sequences were later automatically reannotated by mapping them against the reference genome with the help of exonerate. In case that an existing gene model was replaced by multiple new gene models, we additionally selected one of the new gene models to inherit the WormBase identifier of the old gene model to allow WormBase to record the history of a given gene model. Usually, either the most conserved or the longest new gene model was chosen. Due to the fact that a single artificially fused gene could cause missing homologs and orthologs for multiple *C. elegans* genes, some loci were curated multiple times. We randomly picked some of these cases to compare the classifications and the corresponding corrections from multiple curators, which turned out to be largely consistent. In case of redundant curations, one out of many possible curations for a given locus was chosen based on the following criteria: preference towards higher number of new models, experience of the curator (number of curated loci), and transcriptional evidence over gene prediction.

### Phylostratigraphy and orthology predictions

Outgroup data sets were defined by concatenating all protein sequence data from different species in the ladder-like phylogeny leading to *C. elegans* (Fig. 1). More precisely, we pooled all data from species in an induced subtree defined by branches with roman numbers in Fig. 1. We then ran a BLASTP search (e-value < 0.001) of *C. elegans* proteins (longest isoform per gene) against each of the outgroup data sets. Starting from the *C. elegans* genes with homologs in the most distant outgroup set (VIII), we iteratively defined phylostrata by comparison with the next, more closely related outgroup set. The results of this analysis are summarized in Supplemental Table 1. *C. elegans* specific genes are assigned to phylostratum I, whereas genes that are present in the most divergent outgroups are assigned to phylostratum VIII. Orthologs were defined after performing all pairwise BLASTP searches including self-searches (e-value < 10^−5^) between *C. elegans* and *P. pacificus* and extracting best reciprocal hits from the BLAST output files. Simultaneously, the program orthAgogue was run with default setting on the same input files^48^.

## Supplementary information

Supplementary information

## Data Availability

The strand-specific *de novo* transcriptome was submitted to the European Nucleotide Archive under the accession HAKN01000001^16^ and the Iso-seq data was submitted to the European Nucleotide Archive under the accessions ERX2315712 and ERX2315713^44^. All data sets are also available at http://www.pristionchus.org/download. The initial set of *P. pacificus* gene annotations corresponds to WormBase WS268. Corrections from this study were submitted to WormBase and will be released following curation.
